# Supplementary material for: Spleen Swabs for Sensitive and High-Throughput Detection of African Swine Fever Virus by Real-Time PCR
Source: Viruses. 2024 Aug 18;16(8):1316. doi: 10.3390/v16081316 (PMC11359817; doi:10.3390/v16081316)

Spleen swabs for sensitive and high-throughput detection of African swine fever virus by real-time PCR

Supplemental Material

Supplemental Figure S1. Collecting spleen swab samples: A) Incising the spleen along its length, B) Inserting the swab into the incision before pressing firmly and twisting, C) Ensuring that the swab is fully soaked and, D) Submerging the soaked swab in sterile PBS.

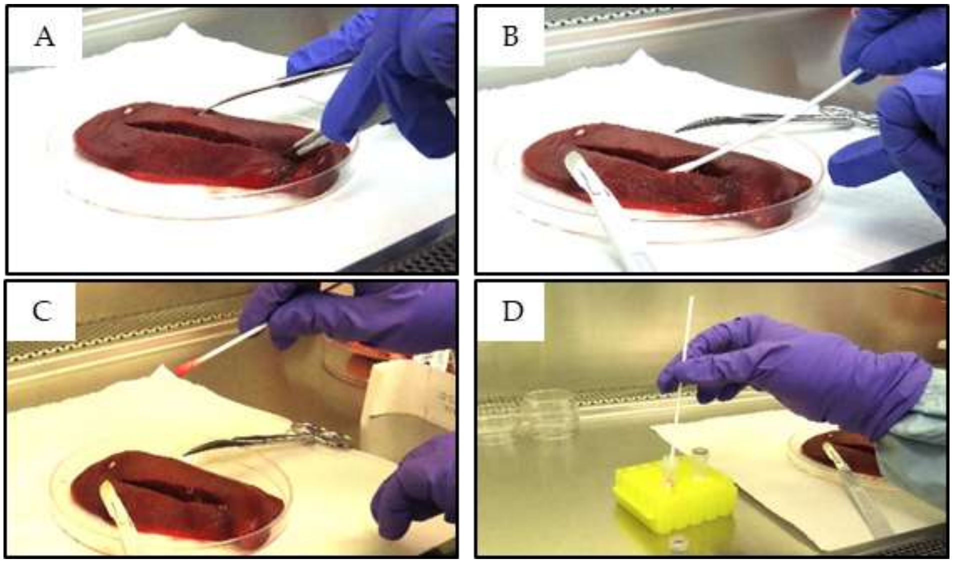

Supplemental Table S1. Real-time PCR results for the 20 known negative samples, 14 wild boar and 6 domestic pig spleen samples collected under the ongoing CanSpot Canadian ASF surveillance system (https://www.animalhealthcanada.ca/canspotasf). ND = not detected.

Strain	Sample	Tignon - ASFV		Zsak - ASFV		Moniwa - B-Actin	
		Homogenate	Swab	Homogenate	Swab	Homogenate	Swab
Healthy Boar	53	ND	ND	ND	ND	23.51	23.83
	54	ND	ND	ND	ND	22.93	23.34
	55	ND	ND	ND	ND	21.51	22.46
	56	ND	ND	ND	ND	21.33	22.29
	57	ND	ND	ND	ND	23.43	23.44
	58	ND	ND	ND	ND	23.34	23.81
	59	ND	ND	ND	ND	21.18	20.48
	60	ND	ND	ND	ND	21.60	21.28
	61	ND	ND	ND	ND	23.01	23.26
	62	ND	ND	ND	ND	21.11	21.60
	63	ND	ND	ND	ND	22.40	23.37
	64	ND	ND	ND	ND	21.24	21.58
	65	ND	ND	ND	ND	21.24	21.84
	66	ND	ND	ND	ND	22.24	20.64
CanSpot	67	ND	ND	ND	ND	22.37	20.87
	68	ND	ND	ND	ND	22.07	21.35
	69	ND	ND	ND	ND	22.84	22.83
	70	ND	ND	ND	ND	22.06	22.14
	71	ND	ND	ND	ND	23.16	22.15
	72	ND	ND	ND	ND	21.62	21.41

Supplemental Table S2. Real-time PCR results for the test for PCR inhibition using Armored RNA Enterovirus spike-in nucleic acids.

Strain	Sample	Armored Entero RNA	
		Homogenate	Swab
ASF Malta 1978	1	27.50	27.79
	2	27.34	27.04
	3	27.45	27.44
	4	27.06	27.22
	5	27.87	26.61
	6	27.85	28.22
	7	27.68	27.66
	8	27.27	27.85
	9	28.10	27.28
	10	27.53	27.15
	11	26.99	27.12
	12	28.21	27.63
	13	27.53	26.82
	14	27.97	27.14
	15	27.30	27.73
ASF Georgia 2007	16	28.21	26.88
	17	27.66	26.69
	18	27.88	26.72
	19	27.66	25.65
	20	27.57	26.93
	21	27.75	27.29
	22	28.12	27.54
	23	28.02	27.60
	24	27.89	27.17
	25	27.20	27.40
	26	27.11	27.04
	27	27.75	27.48
	28	27.49	27.72
	29	28.00	27.85
	30	27.60	26.86
	31	27.46	27.88
	32	27.34	26.56
ASF Estonia 2014	33	36.48	35.58
	34	31.74	30.66
	35	33.29	32.05
	36	35.14	34.05
	37	32.63	32.01

38	31.04	29.36
39	31.50	29.81
40	30.03	29.36
41	29.92	29.27
42	29.46	28.70
43	30.06	29.49
44	30.17	29.54
45	36.40	31.81
46	30.81	29.33
47	32.12	30.26
48	33.99	28.69
49	32.57	29.06
50	29.05	30.12
51	31.05	30.48
52	29.57	29.24

Supplemental Table S3. Results for the inoculation of 11 selected paired homogenate and swab samples into PPL cultures. '+' indicates that HAD were observed within 7 days of inoculation, ND indicates that HAD were not detected.

Strain	Sample	DPI	Homogenate	Swab
ASF Malta '78	8	2	ND	ND
	10	1	ND	ND
	12	1	ND	ND
	13	3	+	+
	14	2	ND	ND
ASF Estonia '14	41	1	ND	ND
	42	2	ND	ND
	43	2	+	+
	44	2	ND	ND
	45	2	ND	+
	46	3	+	+
Ctrl	NA	NA	ND	ND

Supplemental Figure S2. Brightfield image of HAD formation 2 days following inoculation of PPL cultures with paired sample 46 (10X magnification, bar = 100µm).

Sample 46 – Homogenate – Day 2

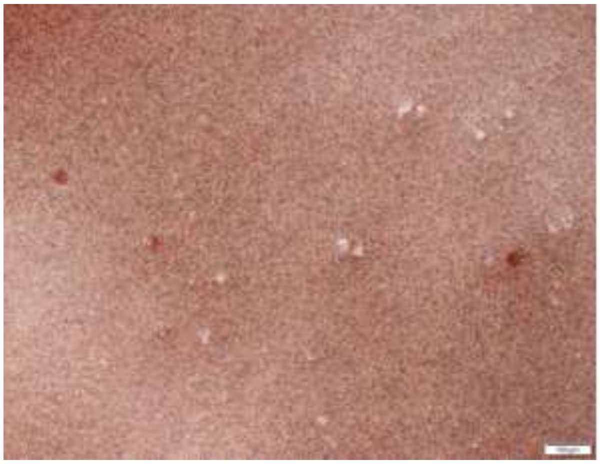

Sample 46 – Swab – Day 2

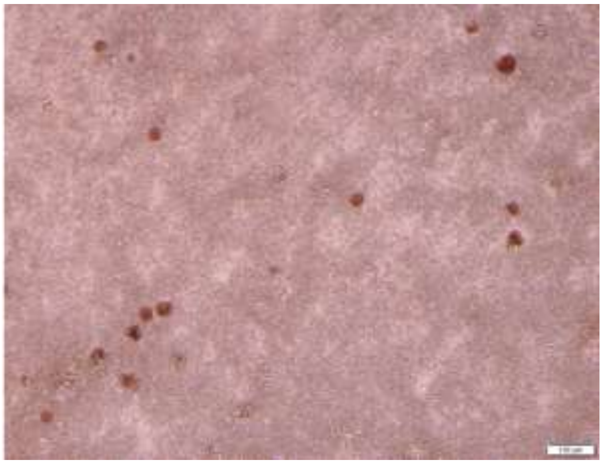

Supplement: Supplementary file 1 [file viruses-16-01316-s001.zip › viruses-3150399-supplementary.pdf]
